# Germacrone protects against oxygen-glucose deprivation/reperfusion injury by inhibiting autophagy processes in PC12 cells

**DOI:** 10.1186/s12906-020-2865-1

**Published:** 2020-03-07

**Authors:** Jianxing Zhang, Li Yuan, Sujie Wang, Jiang Liu, Huiqin Bi, Guojuan Chen, Jingjing Li, Lili Chen

**Affiliations:** 1 Department of Second Neurology, Tangshan Gongren Hospital, No.27 Wenhua Road, Tangshan City, Hebei Province 063000 People’s Republic of China; 2 Department of One Anesthesiology, Tangshan Gongren Hospital, Tangshan City, Hebei Province 063000 People’s Republic of China

**Keywords:** PC12 cells, Oxygen-glucose deprivation/reperfusion, Germacrone, Autophagy, PI3K III/Beclin-1/Bcl-2, PI3K I/Akt/mTOR

## Abstract

**Background:**

Germacrone is an anti-inflammatory ingredient in the Chinese medicine zedoary turmeric. The purpose of this study was to explore the protective mechanism of germacrone against PC12 cells injury caused by oxygen-glucose deprivation/reperfusion (OGD/R).

**Methods:**

OGD/R injury model of PC12 cells was established by using OGD/R (2 h/24 h). The cell viability was assessed by MTT assay and LDH release. The ultrastructure of cells was observed by transmission electron microscopy (TEM). The expression of autophagy related proteins in cells was determined by Western Blot.

**Results:**

The results of ultrastructural observation showed that PC12 cells damaged by OGD/R showed typical autophagy characteristics. In addition, OGD/R observably up-regulated the expression of autophagy related proteins: the class III type phosphoinositide 3-kinase (PI3K III), light chain 3(LC3), and Beclin-1 in PC12 cells, and inhibited the expression of the class I type phosphoinositide 3-kinase (PI3K I), Protein kinase B (Akt), the mammalian target of rapamycin (mTOR), and B-cell lymphoma 2(Bcl-2) proteins. Furthermore, germacrone increased the cell viability of OGD/R-damaged PC12 cells by down-regulating the expression of LC3 protein in cells in a concentration-dependent manner. More importantly, germacrone significantly inhibited the expression of PI3K III, LC3, and Beclin-1 in OGD/R-injured PC12 cells, and up-regulated the expressionof PI3K I, Akt, mTOR, and Bcl-2 proteins in cells, and this inhibited or up-regulated effect was reversed by PI3K I inhibitor (ZSTK474).

**Conclusion:**

The above results indicated that germacrone could inhibit the autophagy effect in OGD/R injury model of PC12 cells, the mechanism of inhibition was regulated by PI3K III/Beclin-1/Bcl-2 and PI3K I/Akt/mTOR pathways, thereby improving the cell viability of PC12 cells and playing a neuroprotective role, which provided a new drug for the treatment of OGD/R.

## Background

According to the regulation of autophagy related genes, autophagy involves the encapsulation of damaged organelles and proteins and then degradation of its contents by lysosomes, so as to form auto-phagosome with double-layer membrane structure and complete the turnover and metabolism of intracellular substances [1–3]. Cerebral hypoxic ischemia/reperfusion (I/R) injury can activate the autophagy effect of neurons [4]. Moderate autophagy induced by mild hypoxia can enhance the ability of nerve cells to resist changes in the external environment, thus improving the survival rate of cells [5]. However, excessive activation of autophagy caused by severe hypoxia can lead to injury and apoptosis of nerve cells [6].

Microtubule-associated protein 1 light chain 3(LC3) exists in two forms: LC3-I and LC3-II. when autophagy is activated, free LC3-I in the cytoplasm can combine with the phosphatidyl ethanolamine (PE) to form the LC3-II [7]. LC3-II of 14 kD is located on the auto-phagosome membrane, and its content is proportional to the number of auto-phagosome vesicles, so LC3-II is one of the marker proteins of auto-phagosome formation [8]. LC3-II/LC3-I ratio is usually adopted to reflect the number of auto-phagosomes and the level of autophagy in cells [9]. Studies have found that PI3K I/Akt/mTOR and PI3K III/Beclin-1/Bcl-2 are important signaling pathways involved in cell autophagy [10, 11]. The activation of PI3K I/Akt/mTOR signaling pathway inhibits autophagy effect in various cells, and activated PI3K I can up-regulate the expression of Akt, and activate the downstream mTOR protein phosphorylation [12]. The mTOR is an important factor related to autophagy, and its activation can effectively block the activation of autophagy effect [13]. In PI3K III/Beclin-1/Bcl-2 signaling pathway, Beclin-1 is a specific autophagy gene, and Beclin-1 protein can form a complex with PI3K III to induce autophagy related proteins to locate in the auto-phagosome membrane, thus promoting the formation of auto-phagosome [14].

Germacrone is one of the active components in zedoary turmeric extract, which has anti-inflammatory, antioxidant and antiviral activities [15, 16]. Feng et al. reported that in the antiviral test in vitro, germacrone showed high anti-porcine reproductive respiratory syndrome virus (PRRSV) infection activity by inhibiting the replication process of PRRSV [17]. Recent studies have found that germacrone has a significant anti-cancer effect, can inhibit the progress of colorectal cancer, liver cancer, breast cancer, lung cancer and other cancers, and has the characteristics of high efficiency and low toxicity [18–20]. Lim et al. found that germacrone inhibited breast cancer progression by inhibiting estrogen receptor α and synergizing with other anti-tumor drugs 5- fluorouracil and methotrexate [21]. However, there are relatively few studies on the neuroprotective effects of germacrone. Previous work found that microglial activation had crucial effects on neuroprotection and neuroinflammatory response, but its over-activation was neurotoxic. For instance, the activation of peroxisome proliferator-activated receptor gamma pathway could induce M2 phenotype of microglia (alternative activation), and promote proliferation and differentiation of neural precursor cells; M2 microglia could reduce inflammatory response with releasing anti-inflammatory cytokines (like IL-10, IL-4 and TGF-β) and then promote neurogenesis and repair [22]. In addition, the anti-inflammatory agents could modulate microglial activation and then provide neuroprotection [23]. Wu et al. investigated the neuroprotective effects of germacrone in rats of transient middle cerebral artery occlusion/reperfusion injury, and showed germacrone could attenuate that injuries [24]. As a result, we speculated that germacrone might play an important role in neuroprotection. In this study, the oxygen-glucose deprivation/reperfusion (OGD/R) model of PC12 cells was established to simulate the hypoxic I/R injury of nerve cells in vitro, in order to explore the protective mechanism of natural compound germacrone on autophagy induction during OGD/R injury of PC12 cells, which would provide a certain theoretical basis for further development and utilization of germacrone.

## Methods

### Materials

**Fig. 1:**
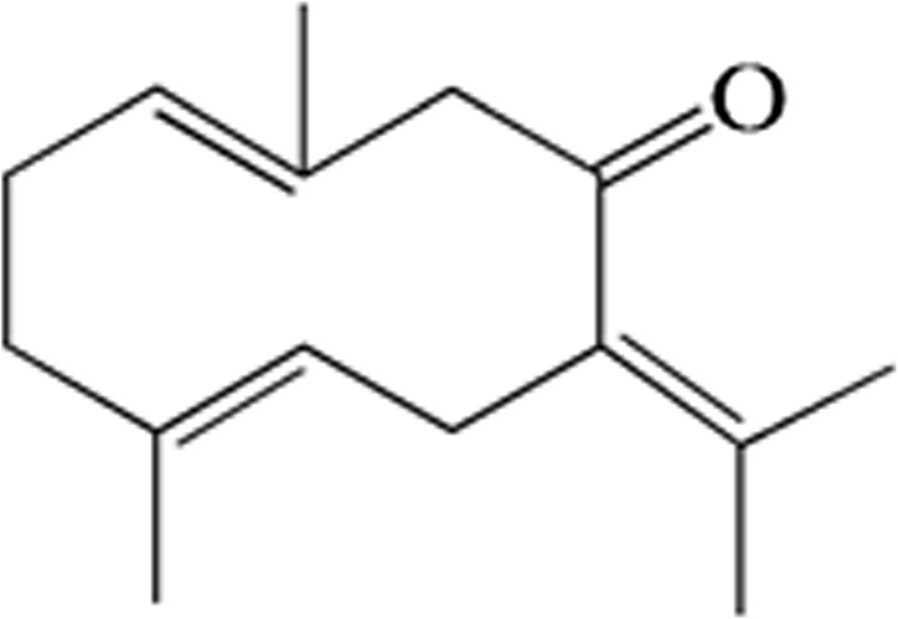
Chemical structure diagram of germacrone (C_15_H_22_O)

Germacrone with purity greater than 98% (molecular weight: 218.34) was purchased from Nanjing puyi biotechnology co., LTD. (Nanjing, China), and its chemical structure was shown in Fig. 1.

### Cell culture

PC12 neurons (rat adrenal pheochromocytoma cells) were obtained from the key laboratory of neurobiology, Shanghai institute of life sciences, Chinese academy of sciences, the number in American Type Culture Collection (ATCC) is CRL-1721. PC12 neurons were cultured using RPMI 1640 medium (Gibco, Paisley, Scotland) containing 7.5% horse serum and 10% Fetal Bovine Serum (Gibco) in an incubator at 37 °C with 5% CO_2_.

### Establishment of PC12 cell OGD/R model

The improved OGD/R method was used to simulate reperfusion injury (I/R) in vitro, and PC12 cell OGD/R model in vitro was established [25]. Briefly, the culture medium (RPMI 1640 medium) was removed and PC12 cells (1 × 10^5^ cells) were washed twice using RPMI 1640 medium (without glucose). PC12 cells were transferred to 37 °C, N_2_/CO_2_ (95%/5%) in a hypoxic incubator chamber (Thermo, USA) for 10 min to reduce the oxygen content of the cells below 1%. Then, RPMI 1640 culture medium (without glucose) was added to the cells for 2 h in the hypoxic incubator chamber, namely OGD phase. Next, cells were cultured in RPMI 1640 medium containing 7.5% horse serum and 10% FBS at air/CO_2_ (95%/5%), namely R phase. The control cells were not treated with OGD/R.

### Experimental grouping

Firstly, PC12 cells were divided into control group and OGD/R groups, and the reperfusion time of OGD/R groups was 0, 3, 6, 12, 24 and 48 h, respectively, so as to determine the optimal reperfusion time of OGD/R model. Next, PC12 cells were treated with 5–80 μM germacrone for 2 h after OGD/R, and 10 μM nimodipine (Bayer healthcare Co., Ltd., Leverkusen, Germany) as a positive control group, to determine the optimal treatment concentration for germacrone. PC12 cells were then treated 30 min before OGD/R with 10 mM PI3K III inhibitor 3-MA (Sigma) and 1 μM PI3K I inhibitor Selleck (ZSTK474) to determine the effect of germacrone on autophagy in PC12 cells. Nimodipine was dissolved in RPMI 1640 medium. The germacrone, 3-MA and ZSTK474 were dissolved in DMSO (Sigma), and the final concentration of DMSO was less than 0.1%.

### Observation of the ultrastructure of cells by TEM

TEM (Hitachi-600, Japan) was used to observe the ultrastructural changes of PC12 cells, so as to evaluate the formation of auto-phagosomes in cells. Simply put, PC12 cells were treated accordingly and collected. Cells were fixed with 2.5% glutaraldehyde and incubated with 1% OsO_4_. Next, cells were dehydrated with gradient ethanol and embedded with Epon 812. Cells were then stained with citric acid and uranium acetate and photographed using TEM.

### Cell viability assay

MTT (Sigma) and LDH kit (Nanjing jiancheng institute of biological engineering) were used to determine the cell viability of PC12 cells. MTT assay was performed as described in the literature [26]. LDH release in cell supernatant was determined with LDH kit, and the specific steps were strictly in accordance with the instructions of the kit.

### Western blot analysis

Western blot analysis was performed as described in the literature [27]. In brief, the total proteins in cells were extracted using cell lysate, and were quantified. The extracted proteins were separated by 10–15% SDS-PAGE and transferred to cellulose acetate membrane. The 5% skim milk powder solution was used to close the membrane for 2 h, then the primary antibody was used to incubate the membrane at 4 °C overnight. The goat IgG with HRP conjugate (Santa Cruz Biotechnology, Santa Cruz, CA, USA) was incubated at room temperature for 1 h. The protein band was then colored and the grayscale value of the protein band was analyzed using ImageJ software. The β-actin (1, 5000, Beijing zhongshan jinqiao biotechnology co., LTD., Beijing, China) was used as an internal control. Among them, mTOR, Akt, PI3K I, Bcl-2, PI3K III, Beclin-1 and LC3 were obtained from Abcam (Cambridge, Massachusetts, USA) with a dilution ratio of 1: 1000.

### Statistical analysis

All data were expressed as mean standard ± deviation (SD). One-way ANOVA and post-test LSD were used for significance analysis, and *P* < 0.05 indicated a significant difference.

## Results

### Effect of OGD/R on cell viability and autophagy in PC12 cells

**Fig. 2:**
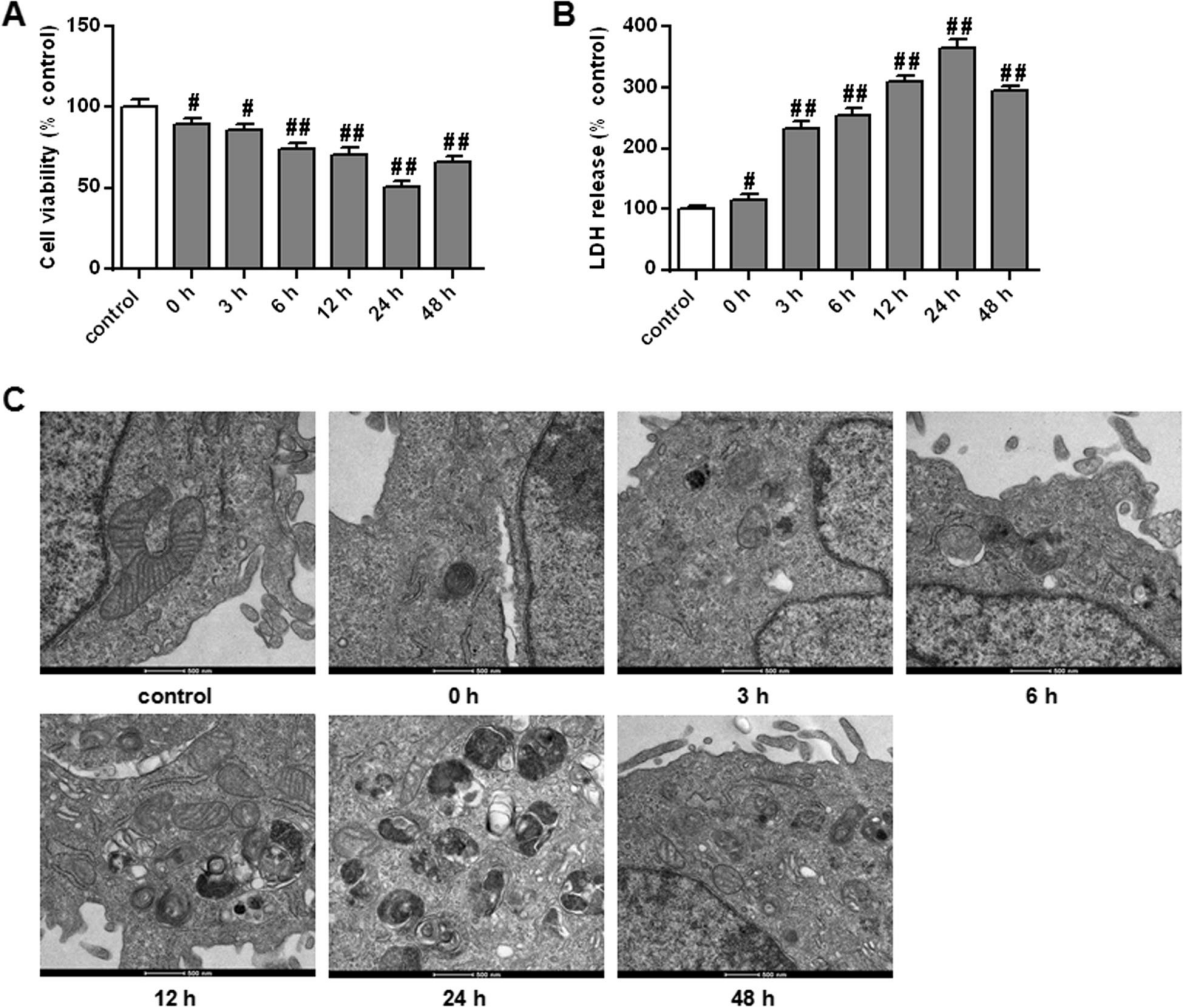
Effect of OGD/R on cell viability and autophagy in PC12 cells (A) Determination of the cell viability of PC12 cells without OGD/R injury and after OGD/R (0, 3, 6, 12, 24 and 48 h) injury by MTT assay. The control group without OGD/R injury. (B) Determination of the LDH release of PC12 cells after OGD/R by kit assay. (C) Observation of the ultrastructural changes of PC12 cells after OGD/R 0 h(b), 3 h(c), 6 h(d), 12 h(e), 24 h(f) and 48 h(g) injury by TEM (× 20,000). Note: *n* = 6; ^#^*P* < 0.05, ^##^*P* < 0.01, vs control group (a)

The cell viability of PC12 cells was determined by MTT assay and toxicity test kit. MTT results showed that with the extension of reperfusion time, the cell viability showed a declining trend and reached the lowest value at 24 h after reperfusion (Fig. 2A, *P* < 0.01). Meanwhile, LDH results showed that with the extension of reperfusion time, the release of LDH in PC12 cells showed an increasing trend, and the highest value was reached at 24 h after reperfusion (Fig. 2B, *P* < 0.01). In addition, we used TEM to observe the ultrastructure of PC12 cells. The results found that the internal structure of cells in the control group was normal, while cells in the OGD/R 0-h group began to show vesicles with auto-phagosome characteristics, indicating that OGD/R had caused the activation of autophagy effect in PC12 cells (Fig. 2C). At the same time, the number of vesicles was the highest in the OGD/R 24 h group, and the typical characteristics of apoptosis and necrosis were observed in cells (Fig. 2C), suggesting that the autophagy effect of PC12 cells induced by OGD/R 24 h was the most significant.

### Effect of OGD/R on autophagy related protein expressions in PC12 cells

**Fig. 3:**
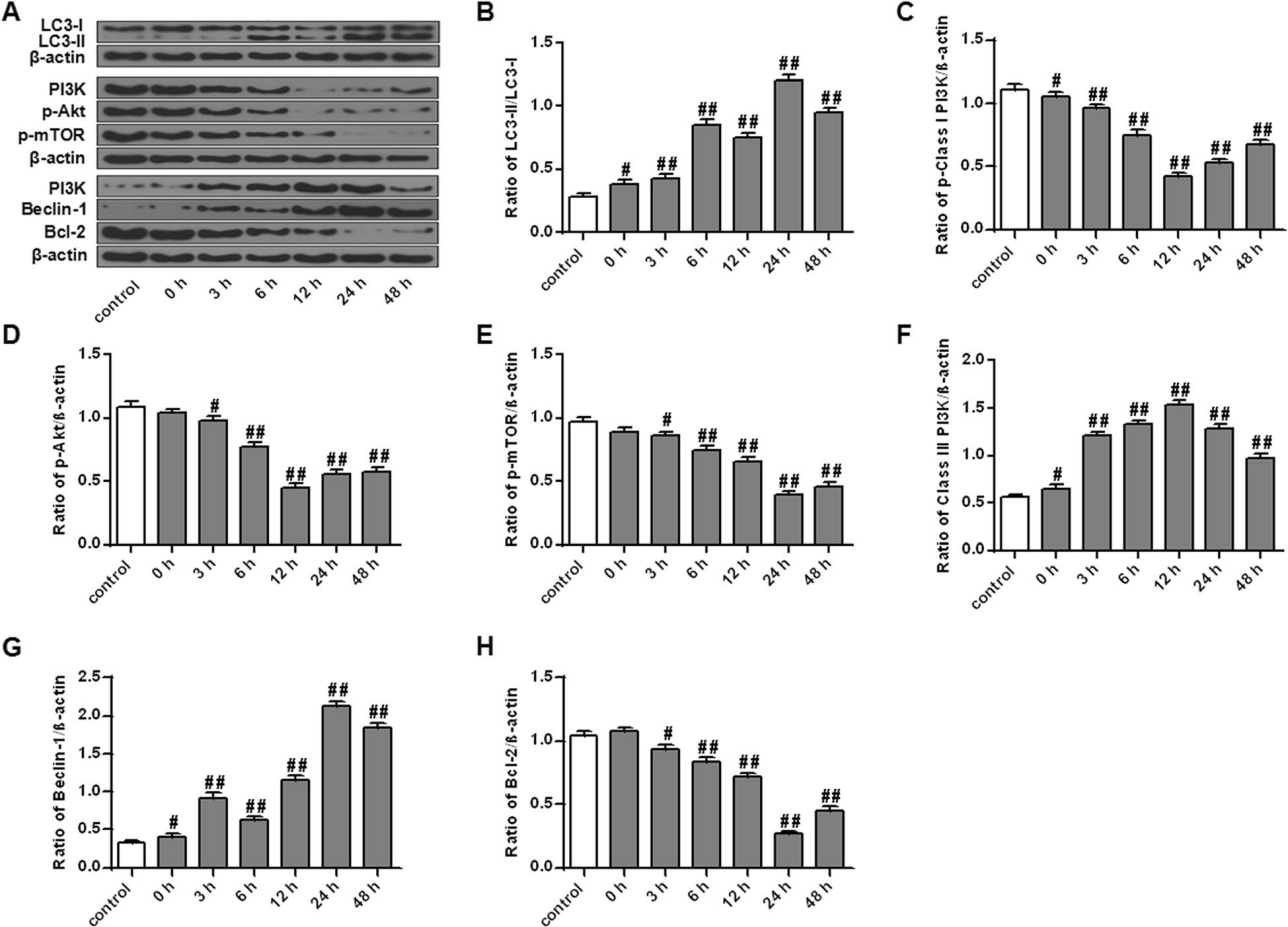
Effect of OGD/R on autophagy related protein expressions in PC12 cells **a** Detection of the protein expressions of LC3, Beclin-1, PI3K III, Bcl-2, PI3K I, p-Akt and p-mTOR in PC12 cells by Western Blot. **b**-**h** Quantitative analysis of the gray value of each protein band. Note: *n* = 3; ^#^*P* < 0.05, ^##^*P* < 0.01, vs control group. p-Class I PI3K: PI3K I protein; p-Akt: Akt protein; p-mTOR: mTOR protein; Class III PI3K: PI3K III protein

LC3 and Beclin-1 are typical markers of autophagy activation [28]. The effect of OGD/R on the expression of LC3 and Beclin-1 in PC12 cells was first determined by Western Blot. The results showed that compared with the control group, the expression of LC3-II/LC3-I and Beclin-1 in cells of the OGD/R 0-h group was remarkably up-regulated (*P* < 0.05), and reached the maximum at 24 h of OGD/R (Fig. 3a, b and g, *P* < 0.01). In addition, we also determined the effect of OGD/R on two autophagy related signaling pathways, PI3K I/Akt/mTOR and PI3K III/Beclin-1/Bcl-2, in PC12 cells. The results found that compared with the control group, OGD/R observably inhibited PI3K I and Akt protein expressions in cells and reached the lowest level at 12 h after OGD/R (Fig. 3a, c and d, *P* < 0.01). Meanwhile, the protein expression of mTOR in cells with OGD/R for 3 h began to be significantly down-regulated (*P* < 0.05), and reached the minimum at 24 h after OGD/R (Fig. 3a and e, *P* < 0.01). Furthermore, OGD/R remarkably upregulated the protein expression of PI3K III in cells and reached a maximum at 12 h after OGD/R (Fig. 3a and f, *P* < 0.01). Simultaneously, the protein expression of Bcl-2 began to be markedly down-regulated in the OGD/R 3 h group (*P* < 0.05), and reached the minimum at 24 h after OGD/R (Fig. 3a and h, *P* < 0.01). Based on the above experimental results, 24 h of OGD/R was selected for the following experimental research.

### Effects of different concentrations of germacrone on PC12 cell viability

**Fig. 4:**
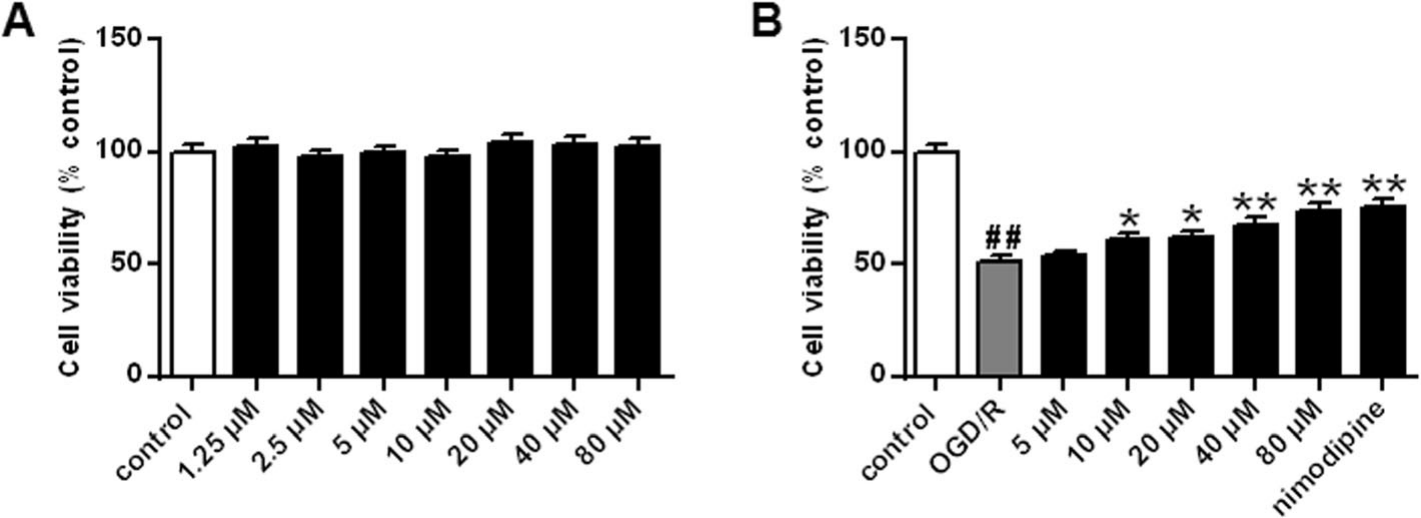
Effect of different concentrations of germacrone on PC12 cell viability **a** Detection of the effect of 1.25–80 μM germacrone on PC12 cells viability by MTT assay. **b** Detection of the effect of germacrone on PC12 cells viability after OGD/R injury by MTT assay. Note: *n* = 6; ^##^*P* < 0.01, vs control group; ^*^P < 0.05, ^**^P < 0.01, vs OGD/R group

First, No OGR/R treated PC12 cells were treated with germacrone at 1.25–80 μM for 24 h, and the activity of PC12 cells was determined by MTT assay. The results showed that, compared with the control group (not treated with germacrone or OGR/R injury), germacrone at different concentrations had no significant effect on the cell viability of PC12 cells (Fig. 4a, *P* > 0.05), indicating that germacrone had no toxic effect on PC12 cells. In addition, we also determined the effect of different concentrations of germacrone (1.25–80 μM) on the activity of PC12 cells after the OGD/R 24 h injury. The results found that the cell viability of PC12 cells in the OGD/R 24 h group was remarkably lower than that in the control group (*P* < 0.01), while that in germacrone groups (10–80 μM) were significantly higher than that in the OGD/R 24 h group (Fig. 4b, *P* < 0.05, *P* < 0.01). The protective effect of 80 μM germacrone treatment group was similar to that of the positive control drug nimodipine. The above results showed that germacrone (10–80 μM) could observably up-regulate the cell viability of PC12 cells after OGD/R 24 h injury. In subsequent experiments, PC12 cells were treated with 20, 40, and 80 μM germacrone.

### Germacrone alleviated OGD/R-induced PC12 cell injury by inhibiting autophagy

**Fig. 5:**
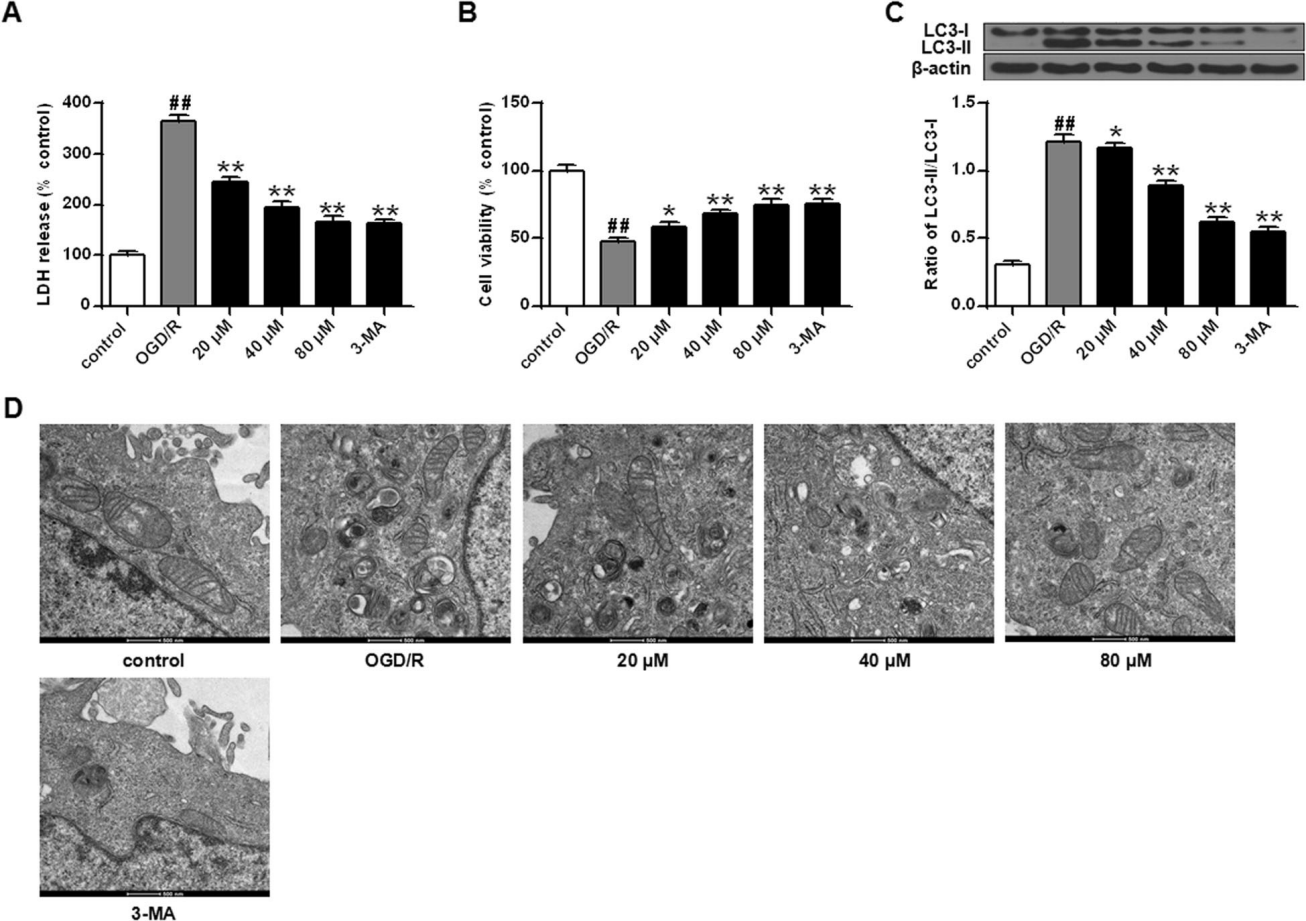
Germacrone alleviated OGD/R-induced PC12 cells injury by inhibiting autophagy (A) Detection of the LDH release in each group by kit assay (*n* = 6). (B) Detection of the cell viability of each group by MTT assay (*n* = 6). (C) Detection of the expression of LC3 in cells of each group by Western Blot (*n* = 3). (D) Observation of the ultrastructural of PC12 cells without OGD/R injury(a) and after OGD/R 24 h injury(b), as well as the ultrastructural of PC12 cells treated with 20 μM (c), 40 μM (d), 80 μM(e) of germacrone and 3-MA(f) after OGD/R 24 h injury by TEM (× 20,000). Note: ^##^*P* < 0.01, vs control group; ^*^P < 0.05, ^**^P < 0.01, vs OGD/R group

First, PC12 cells were treated with 10 mM 3-MA or 20–80 μM germacrone to investigate the effect of germacrone on autophagy and cell viability in PC12 cells after OGD/R 24 h injury. LDH assay results showed that the LDH release of PC12 cells treated with 3-MA and germacrone was observably lower than that of OGD/R 24 h group (Fig. 5A, *P* < 0.01). Furthermore, MTT assay showed that the cell viability of PC12 cells treated with 3-MA and germacrone was remarkably higher than that of OGD/R group (Fig. 5B, *P* < 0.05, *P* < 0.01). In addition, Western Blot results showed that the expression of LC3-II/LC3-I in cells of the 3-MA and germacrone treatment groups were markedly lower than that of the OGD/R 24 h group (Fig. 5C, *P* < 0.05, *P* < 0.01), which suggested that 3-MA or germacrone effectively blocked the autophagy activation of PC12 cells induced by OGD/R 24 h injury. Besides, in order to verity the effect of germacrone in PC12 cells after OGD/R 24 h injury, we observed the ultrastructural changes in each group by TME. The results showed OGD/R induced some auto-phagosome in cells, while the introduction of 3-MA or germacrone inhibited the formation of auto-phagosome vesicles. Meanwhile, the number of vesicles tended to decrease after treated with 20, 40 and 80 μM germacrone, and the group treated with 80 μM germacrone showed similar characteristics with the 3-MA and control group (Fig. 5D).

### The molecular mechanism of germacrone blocked OGD/R-induced autophagy in PC12 cells

**Fig. 6:**
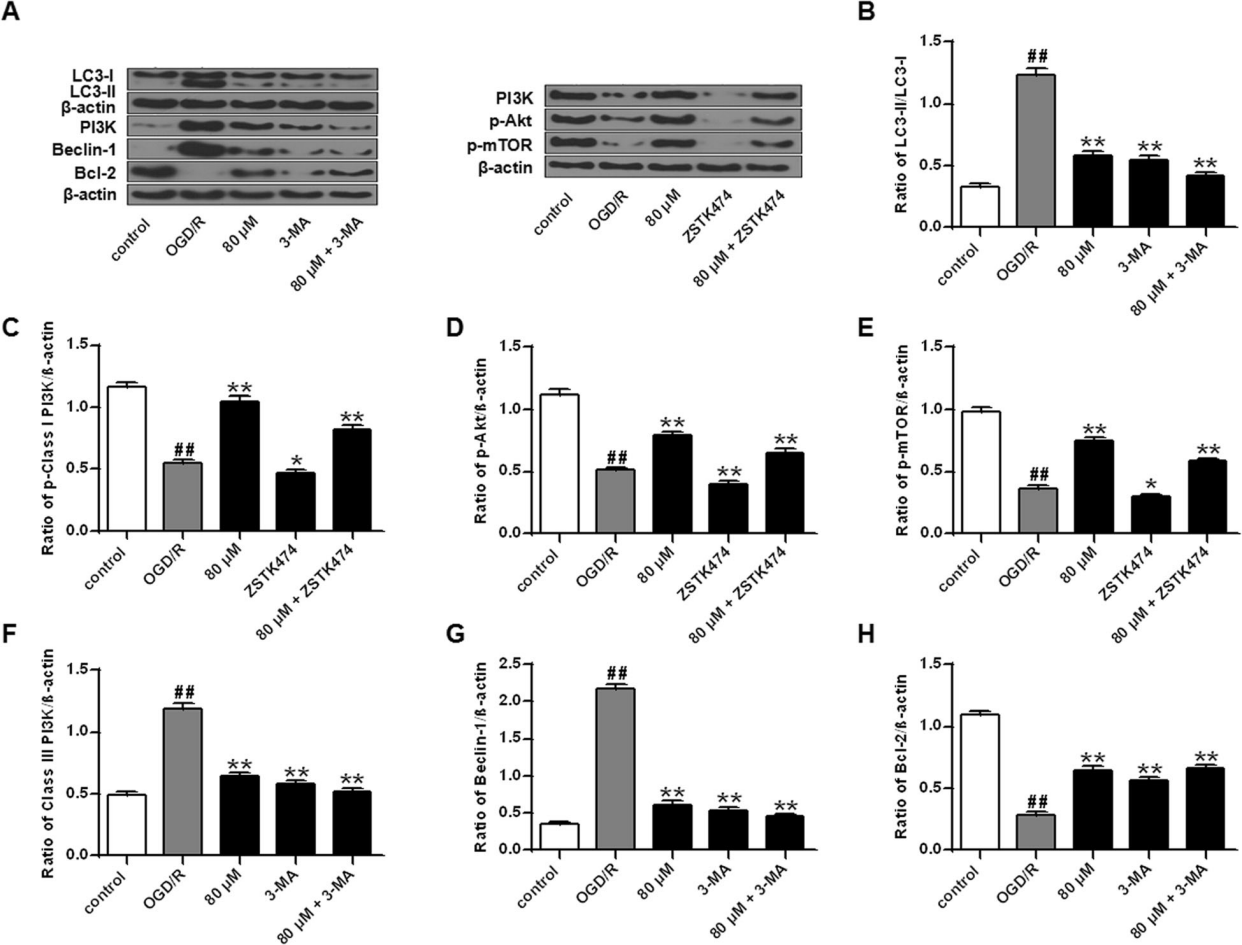
The molecular mechanism of germacrone blocked OGD/R-induced autophagy in PC12 cells (A) Compare the effect of treatment with PI3K III inhibitor (3-MA)(a) and PI3K I inhibitor (ZSTK474)(b). Detection of the protein expression of LC3, Beclin-1, PI3K III, Bcl-2, PI3K I, p-Akt and p-mTOR in PC12 cells by Western Blot. (B-H) Quantitative analysis of the gray value of each protein band. Note: *n* = 3; ^##^*P* < 0.01, vs control group; ^*^P < 0.05, ^**^P < 0.01, vs OGD/R group. p-Class I PI3K: PI3K I protein; p-Akt: Akt protein; p-mTOR: mTOR protein; Class III PI3K: PI3K III protein

In order to explore the mechanism of germacrone blocking autophagy in PC12 cells induced by OGD/R 24 h injury, the expression of autophagy related proteins in PC12 cells was determined by Western Blot. The results showed that compared with the OGD/R 24 h group, the expression of LC3-II/LC3-I, PI3K III and Beclin-1 in the germacrone and/or 3-MA treatment groups was markedly down-regulated (Fig. 6A, B, F and G, *P* < 0.01), while the expression of Bcl-2 was significantly up-regulated (Fig. 6A, H, *P* < 0.01). In addition, compared with the OGD/R 24 h group, the expression of PI3K I, Akt and mTOR in the germacrone treatment groups was significantly increased (*P* < 0.01), and germacrone also alleviated the inhibitory effect of ZSTK474 on the expression of these proteins (Fig. 6A, C, D and E, *P* < 0.01). The above results suggested that germacrone blocked the activation of OGD/R 24 h induced autophagy in PC12 cells by regulating the PI3K III/Beclin-1/Bcl-2 and PI3K I/Akt/mTOR pathways.

## Discussion

PC12 cell lines, derived from rat adrenal medulla pheochromocytoma, can secrete catecholamine transmitters and have sympathetic neuron characteristics, so they have been widely used in vitro to study of neuron injury mechanism [29, 30]. Studies have found that severe cerebral hypoxic I/R injury can induce autophagic death of neurons [31, 32]. At present, OGD/R model can simulate cerebral ischemia and hypoxia in vivo, which is a classic and mature model for in vitro ischemia and hypoxia research [33]. In this method, the concentration of CO_2_ and O_2_ in the incubator was precisely controlled by direct instillation of N_2_, and the glucose in the cell culture medium was deprived at the same time, so as to create a hypoxic and hypoxic environment for cultured cells and simulate the situation of cerebral ischemia and hypoxia in vivo [34]. MTT assay is a classical method to detect cell survival and growth, and LDH release can reflect the degree of cell necrosis [35].

LC3-II is one of the marker proteins for the formation of auto-phagosomes, and the ratio of LC3-II/LC3-I is usually used to reflect the number of auto-phagosomes and the level of autophagy in cells [36]. Mo et al. found that after OGD/R injury, the expression of autophagy related protein LC3-II in PC12 cells, was markedly increased, and the cell survival rate was significantly reduced [37]. In the present study, it was found that the expression of LC3-II/LC3-I in cells treated with OGD/R was remarkably up-regulated and reached the maximum at 24 h after OGD/R treatment, suggesting that OGD/R might activate the autophagy effect in PC12 cells. It has been found that PI3K I/Akt/mTOR and PI3K III/Beclin-1/Bcl-2 are important signaling pathways involved in cell autophagy [38, 39]. This study found that OGD/R could observably inhibit the expression of PI3K I, Akt and mTOR, which indicated that OGD/R could repress the PI3K I/Akt/mTOR pathway to promote the autophagy effect of PC12 cells. Meanwhile, OGD/R could markedly increase the protein expression of PI3K III and Beclin1, and down-regulate the expression of Bcl-2, which suggested that OGD/R could induce the autophagy effect of PC12 cells through the activation of PI3K III/Beclin-1/Bcl-2 pathway. Based on the above experimental results, 24 h of OGD/R was selected for the following experimental research.

Germacrone has anti-inflammatory, antioxidant, antiviral and anti-tumor and other physiological activities [40–42]. The role of germacrone has attracted our attention in the OGD/R-damaged PC12 cells. In this study, the OGD/R model of PC12 cells was established to explore the protective mechanism of germacrone on the induction of autophagy during OGD/R 24 h injury. The results showed that germacrone (10–80 μM) could remarkably up-regulate the cell viability of PC12 cells after OGD/R injury, and 20, 40 and 80 μM germacrone were selected to treat PC12 cells in follow-up experiments. Classic autophagy upstream inhibitor 3-MA can inhibit the activity of PI3K III type, thus inhibiting the formation of autophagy body [43]. The results of this study showed that 3-MA or 80 μM germacrone effectively inhibited the expression of autophagy related proteins in OGD/R-induced PC12 cells, thereby improving the cell viability of PC12 cells after OGD/R injury. In addition, this study also found that OGD/R and PI3K I inhibitor ZSTK474 observably inhibited the PI3K I/Akt/mTOR pathway, and 80 μM germacrone remarkably alleviated the inhibitory effect. Chang et al. found that ganoderic acid A could alleviate the autophagy effect of rat neural stem cells activated by hypoxia injury by activating the PI3K I/AKT/mTOR pathway [44]. Huang et al. reported that the combination of Ginsenoside Rg1 and Astragaloside IV inhibited OGD/R-induced autophagy in PC12 cells by regulating PI3K III/Beclin-1/Bcl-2 and PI3K I/Akt/mTOR pathways [45]. Therefore, the results of this study suggested that germacrone inhibited the activation of OGD/R-induced autophagy in PC12 cells by blocking the PI3K III/Beclin-1/Bcl-2 pathway and activating the PI3K I/Akt/mTOR pathway.

In addition, in recent studies, the main limitation of how to deal with autophagy due to nerve injury is the lack of vivo experiments. In recently published work, it has been reported that modulation of microglial activation with anti-inflammatory agents can provide neuroprotection [22, 23]. Since germacrone contains anti-inflammatory ingredients, Therefore, dose germacrone have neuroprotective effect also because of its anti-inflammatory property? The underlying mechanism needs further investigation.

## Conclusion

In conclusion, the present study assessed the neuroprotective mechanism of germacrone against OGD/R-induced PC12 cells injury. These results indicated that germacrone could inhibit the autophagy effects by regulating the autophagy related proteins pathways, including PI3K III/Beclin-1/Bcl-2 and PI3K I/Akt/mTOR pathways. So, germacrone could improve viability of PC12 cells and played a neuroprotective role, which provided a certain theoretical basis for further development of germacrone.

## Data Availability

The analyzed data sets generated during the study are available from the corresponding author on reasonable request.

## Abbreviations

Akt: Protein kinase B
Bcl-2: B-cell lymphoma 2
I/R: Ischemia/reperfusion
LC3: Light chain 3
mTOR: The mammalian target of rapamycin
OGD/R: Oxygen-glucose deprivation/reperfusion
PI3K I: The class I type phosphoinositide 3-kinase
PI3K III: The class III type phosphoinositide 3-kinase
TEM: Transmission electron microscopy
